# Concomitant myocarditis and painless thyroiditis after AstraZeneca coronavirus disease 2019 vaccination: a case report

**DOI:** 10.1186/s13256-022-03438-z

**Published:** 2022-05-17

**Authors:** Apichai Marsukjai, Nonthikorn Theerasuwipakorn, Monravee Tumkosit, Pairoj Chattranukulchai, Panudda Srichomkwun, Somchai Prechawat

**Affiliations:** 1 Division of Cardiovascular Medicine, Department of Medicine, Faculty of Medicine, Chulalongkorn University, Cardiac Center, King Chulalongkorn Memorial Hospital, Bangkok, 10330 Thailand; 2 Department of Radiology, Faculty of Medicine, Chulalongkorn University, King Chulalongkorn Memorial Hospital, Bangkok, Thailand; 3 Division of Endocrinology and Metabolism, Department of Medicine, Faculty of Medicine, Chulalongkorn University, King Chulalongkorn Memorial Hospital, Bangkok, Thailand; 4 Excellent Center in Diabetes, Hormones and Metabolism, King Chulalongkorn Memorial Hospital, Thai Red Cross Society, Bangkok, Thailand

**Keywords:** COVID-19, AstraZeneca COVID-19 vaccine, Myocarditis, Thyroiditis, Case report

## Abstract

**Background:**

Incidence of myocarditis following messenger RNA coronavirus disease 2019 vaccination has been widely described, but this clinical scenario after adenoviral vector coronavirus disease 2019 vaccination has only been rarely reported. In addition, a few case reports of thyroiditis after adenoviral vector coronavirus disease 2019 vaccination have been published.

**Case presentation:**

A 55-year-old Thai woman presented with palpitation without neck pain 14 days after receiving AstraZeneca coronavirus disease 2019 vaccination. Electrocardiography revealed sinus tachycardia. Her blood tests showed elevation of cardiac troponin and free triiodothyronine with suppressed serum thyroid stimulating hormone, reflecting a hyperthyroid status. Evidence of myocardial inflammation and necrosis from cardiac magnetic resonance imaging supported the diagnosis of recent myocarditis. Laboratory results and imaging findings were consistent with thyroiditis. After 3 weeks of symptomatic treatment, her symptom and blood tests had returned to normal.

**Conclusions:**

This case demonstrates that the adenoviral vector coronavirus disease 2019 vaccine could possibly cause myocarditis and painless thyroiditis. Clinicians should have a high index of suspicion and promptly evaluate these conditions, despite minimal symptoms.

**Supplementary Information:**

The online version contains supplementary material available at https://doi.org/10.1186/s13256-022-03438-z.

## Background

Since the emergence of the coronavirus disease 2019 (COVID-19)/severe acute respiratory syndrome coronavirus 2 (SARS-CoV-2) pandemic, effective vaccines against COVID-19 have been developed [1]. The AstraZeneca COVID-19 vaccine (ChAdOx1-S/nCoV-19 [recombinant], AZD1222), a replication-deficient adenoviral vector vaccine that contains the SARS-CoV-2 spike protein gene, triggers the immune system to generate an immune response against the coronavirus and retain that information in memory immune cells [2, 3]. This vaccine was used for mass vaccination nationally and globally. In Thailand, more than 40 million doses have already been given. The most well-publicized adverse reactions are vaccine-induced thrombosis and thrombocytopenia [4]. However, there are a few published reports regarding thyroiditis and myocarditis following exposure to the AstraZeneca COVID-19 vaccine.

We report the case of a patient who developed concomitant myocarditis and painless thyroiditis after receiving the AstraZeneca COVID-19 vaccine.

## Case presentation

**Fig. 1:**
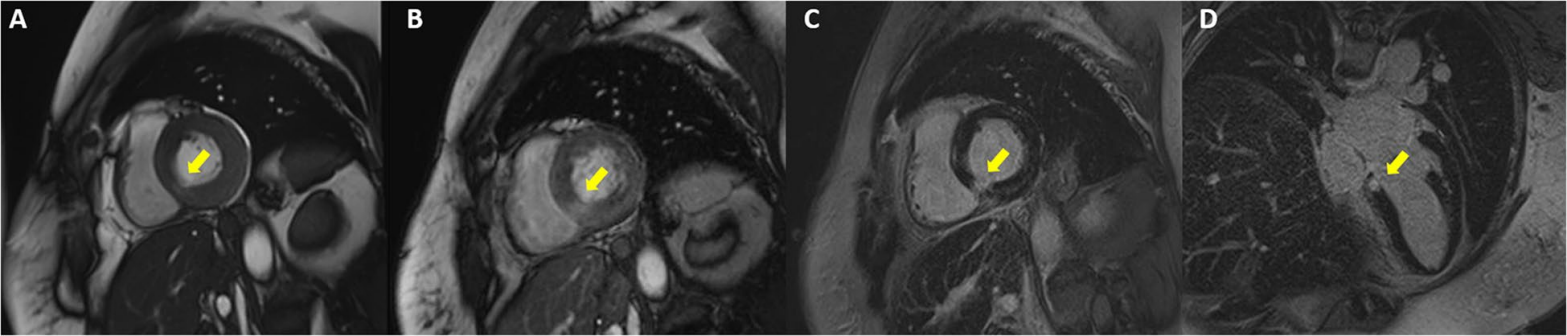
Cardiac magnetic resonance imaging with conventional techniques showed multiple signs of acute focal myocarditis at the basal inferoseptal segment of the left ventricle (arrows). **A** Steady-state free precession (SSFP) cine image in basal short-axis (SAX) view demonstrating regional wall motion abnormality. **B** Early gadolinium enhancement image in basal SAX view revealing hyperenhancement, a sign of myocardial hyperemia. **C**, **D** Delayed gadolinium enhancement images in basal SAX and modified four-chamber views depicting hyperenhancement, a sign of myocardial necrosis and fibrosis

**Fig. 2:**
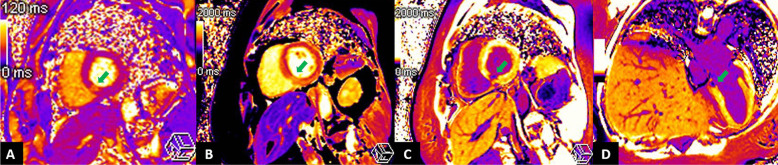
Cardiac magnetic resonance imaging with parametric mapping techniques showed **A** myocardial edema in T2 mapping image, **B** increased T1 relaxation time in native T1 mapping, and **C**, **D** shortened T1 relaxation time in post-contrast T1 mapping

A 55-year-old Thai woman presented with palpitation 14 days after receiving the second dose of the AstraZeneca COVID-19 vaccination. She had no history of fever, dyspnea, chest pain, neck pain, or weight loss. Her past medical histories were essential hypertension and hypercholesterolemia. She had no history of thyroid disease. On admission, she had tachycardia with a heart rate of 120/min. Other examinations including respiratory rate, body temperature, and thyroid gland were unremarkable. Twelve-lead electrocardiography (ECG) revealed sinus tachycardia without evidence of ST–T segment change. Plain chest radiography showed a normal cardiothoracic ratio and pulmonary vasculature. High-sensitivity cardiac troponin I was 2007.5 ng/L (normal < 15.6 ng/L). Thyroid function tests showed elevated serum free triiodothyronine of 7.37 pg/mL (normal 1.60–4.00 pg/mL), normal serum free thyroxine of 1.08 ng/dL (normal 0.70–1.48 ng/dL) with suppressed serum thyroid stimulating hormone (TSH) of 0.113 uIU/mL (normal 0.350–4.940 uIU/mL). Thyroid peroxidase, thyroglobulin, and TSH receptor antibodies were negative (Table 1). High-sensitivity C-reactive protein (4.09 mg/L) and erythrocyte sedimentation rate (11 mm/h) were normal. Nasopharyngeal swab polymerase chain reaction tests for respiratory viruses including COVID-19 were negative. Thyroid ultrasound revealed normal thyroid gland size, homogeneous parenchyma without increased Doppler flow. Iodine-131 uptake study showed very low thyroid uptake (3.95% and 4.39% at 3 and 4 hours, respectively), consistent with thyroiditis. Transthoracic echocardiography (TTE) showed normal biventricular size and function. Cardiac magnetic resonance imaging (CMR) demonstrated basal inferoseptal segment hypokinesia on steady-state free precession (SSFP) cine images (see Additional file 1) with evidence of myocardial edema on T2 mapping (64 ms, normal myocardium 41 ms), myocardial hyperemia on early gadolinium enhancement images, as well as myocardial necrosis on delayed gadolinium enhancement images, native T1 mapping (1511 ms, normal myocardium 1229 ms), and postcontrast T1 mapping (320 ms, normal myocardium 536 ms) (Figs. 1, 2). CMR findings were suggestive of recent myocarditis according to the Lake Louise criteria. Endomyocardial biopsy was omitted because of mild symptoms. After 3 weeks of symptomatic treatment with low-dose beta-blocker (propranolol 30 mg/day) and exercise restriction, her symptom and blood tests had returned to normal (Table 1).

**Table 1:** Blood test results at baseline and follow-up at third week

Blood test	Reference	Baseline	Third week
High-sensitivity troponin I (ng/L)	< 15.6	2007.5	4.1
High-sensitivity C-reactive protein (mg/L)	0–5.00	4.09	0.82
Erythrocyte sedimentation rate, ESR (mm/h)	0–28	11	–
Free triiodothyronine, FT3 (pg/mL)	1.60–4.00	7.37	2.39
Free thyroxine, FT4 (ng/dL)	0.70–1.48	1.08	0.93
Thyroid stimulating hormone, TSH (uIU/mL)	0.350–4.940	0.113	2.333
Anti-thyroglobulin (IU/mL)	< 115	12.90	–
Anti-thyroid peroxidase (IU/mL)	< 34	< 9.0	–
TSH receptors antibody (IU/L)	0–1.75	1.70	–

## Discussion

Myocarditis and pericarditis are well-known potential adverse reactions after mRNA-1273 and BNT162b2 vaccine administration [5]. They are not widely recognized as possible adverse reactions of AstraZeneca COVID-19 vaccine, though the incidence of suspected myocarditis–pericarditis following mRNA and AstraZeneca COVID-19 vaccine from the vaccine adverse event reporting system was similar (1.6–5.0 versus 2.0–3.7 per million doses, respectively) [6]. To date, only a few cases of myocarditis following exposure to the AstraZeneca COVID-19 vaccine have been published [7, 8]. Our patient age was not common for mRNA-induced myocarditis, but the typical age of AstraZeneca COVID-19 vaccine-related myocarditis has not previously been concluded. Vaccine-related myocarditis usually presented with mild symptoms, which resolved spontaneously with conservative treatment [7, 9]. A severe form presented with cardiogenic shock and need for hemodynamic support had been scarcely reported [8, 10]. Chest pain is the most common presentation; unlike other reports, our case presented with palpitation, which could be a manifestation of myocarditis, thyroiditis, or both [9]. CMR is recommended, in addition to ECG, cardiac markers, and TTE, for myocarditis with minimal symptoms due to noninvasiveness and trustworthy tissue characterization ability [11]. This case highlights the significance of CMR for diagnosis of myocarditis, particularly when the presentation is mild and TTE findings are negative.

Post-vaccination thyroiditis, a well-knwon autoimmune/inflammatory syndrome induced by adjuvants (ASIA), has been reported after various types of vaccine, including all COVID-19 vaccine platforms. This condition predominantly affects women in an age range from 26 to 75 years [12–14]. Symptoms, typically mild and self-resolving without specific treatment, can occur 4–21 days after vaccination. Most patients have neck pain at the onset, while only one case of painless thyroiditis, as in our patient, has been reported [13]. At the onset of presentation, patients can have hyperthyroid (most common), hypothyroid, or euthyroid status [13]. Differential diagnoses of post-vaccination thyrotoxicosis included Graves’ disease, and co-occurrence of subacute thyroiditis and Graves’ disease was also reported [15]. Thyroid antibodies and iodine-131 uptake should be investigated to clarify the etiology of thyrotoxicosis.

AstraZeneca COVID-19 vaccine contains recombinant replication-deficient chimpanzee adenovirus vector encoding the SARS CoV-2 spike protein. Possible mechanisms for post-vaccination myocarditis and thyroiditis are molecular mimicry between SARS CoV-2 spike protein and self-antigens (myocyte protein and thyroid peroxidase) as well as triggering of preexisting dysregulated immune pathways [9, 16, 17]. However, the entire mechanism is unclear.

To our knowledge, this is the first case report of concomitant myocarditis and painless thyroiditis following AstraZeneca COVID-19 vaccine administration. The true association cannot be established, although we demonstrate the temporal relationships between vaccine and these conditions.

## Conclusion

This case demonstrates that the adenoviral vector COVID-19 vaccine could possibly cause myocarditis and painless thyroiditis. Clinicians should have a high index of suspicion and promptly evaluate these conditions. Given a low incidence and minimal symptoms in most cases, the COVID-19 vaccine is recommended in the pandemic situation because the benefit from the vaccine greatly outweighs the risks.

## Supplementary Information

**Additional file 1.** SSFP cine image in basal short-axis (SAX) view demonstrating regional wall motion abnormality.

## Data Availability

The data for this case report are located at King Chulalongkorn Memorial Hospital, Bangkok, Thailand.

## Abbreviations

CMR: Cardiac magnetic resonance imaging
COVID-19: Coronavirus disease 2019
ECG: Electrocardiography
GRE: Spoiled gradient echo
SAX: Short-axis
SSFP: Steady-state free precession
TTE: Transthoracic echocardiography
TSH: Thyroid stimulating hormone
